# From Effect Sizes to Clinical Probabilities: An Exploratory Methodological Framework for Evidence Interpretation and Communication

**DOI:** 10.7759/cureus.113119

**Published:** 2026-07-21

**Authors:** Eisuke Iwamoto, Kentaro Murase

**Affiliations:** 1 Department of Integrative Medicine, Acupuncture and Massage Clinic ReLife, Miyakonojo, JPN; 2 Department of Sports Acupuncture and Moxibustion, Kyushu University of Nursing and Social Welfare, Tamana, JPN

**Keywords:** acupuncture, clinical interpretation, effect size, evidence interpretation, minimal clinically important difference, odds ratio, patient communication, probability, therapeutic leverage ratio, treatment outcome

## Abstract

Introduction: Effect sizes such as mean differences (MDs) and standardized mean differences (SMDs) are widely used to summarize treatment effects. However, these measures do not directly indicate the likelihood of clinically meaningful improvement and may be difficult to interpret in clinical practice. This study presents an exploratory methodological framework for re-expressing effect sizes as clinically meaningful probabilities and examines whether these probabilities can support the interpretation and communication of evidence.

Methods: We applied the proposed framework to published effect sizes from representative acupuncture randomized controlled trials and meta-analyses. Assuming normal distributions, MDs and SMDs were re-expressed as probabilities of improvement, worsening, and stability using minimal clinically important difference (MCID) thresholds. These probabilities were further expressed using odds ratios (ORs), therapeutic leverage ratios (TLRs), and absolute probability differences (ΔP). Sensitivity analyses were performed to examine the influence of MCID thresholds, effect sizes, baseline probabilities, and distributional assumptions on probability-based interpretations.

Results: Previously reported MDs and SMDs could be re-expressed as probabilities of improvement, worsening, and stability. ORs, TLRs, and ΔP provided different perspectives on the same probability changes. Sensitivity analyses showed that absolute probabilities varied across assumptions, whereas the overall three-category structure of improvement, worsening, and stability was preserved across all scenarios.

Conclusion: Re-expressing effect sizes as clinical probabilities may provide a more intuitive interpretation of population-level evidence. This framework enables the interpretation of improvement, worsening, and stability simultaneously from a single effect size using complementary probability metrics. Although not intended for individual prediction, it may support evidence interpretation when patient-specific information is still limited. In routine clinical practice, however, evidence should ultimately be applied in conjunction with patient-specific information obtained through history taking and clinical examination. Future prospective studies using individual patient data are needed to validate this framework and evaluate its clinical applicability.

## Introduction

The initial consultation is often characterized by uncertainty regarding treatment outcomes. Patients want to know how much they are likely to improve, whereas clinicians may find it difficult to provide a clear answer when patient-specific information is limited. This challenge is also encountered across many therapeutic interventions, including acupuncture. Under such circumstances, clinicians often rely on average treatment effects derived from clinical evidence. However, effect sizes describe differences between intervention and control groups and do not directly indicate the degree of improvement that patients may expect. As a result, their clinical meaning may be difficult to interpret [1]. Previous studies have shown that the presentation format of treatment effects influences understanding and decision-making, with probability and absolute-risk-based formats often improving interpretation compared with relative measures [2,3].

To address this issue, probability-based interpretations using the minimal clinically important difference (MCID) have been proposed [4]. The MCID functions as a threshold representing a level of improvement that patients can perceive as meaningful and allows effect sizes to be re-expressed as probabilities of improvement [5]. Previous studies have primarily focused on probabilities of clinically meaningful improvement based on MCID thresholds, whereas worsening outcomes have received less attention. We were unable to identify reports that simultaneously examined the probabilities of improvement, stability, and worsening derived from the same effect size. In addition, treatment effects can be expressed using absolute probability differences, relative benefit measures, or odds ratios. Each metric has distinct characteristics, but its respective roles in evidence interpretation and patient communication remain incompletely understood.

To explore whether this framework could facilitate clinical interpretation, we applied it to published effect sizes from acupuncture research. Specifically, improvement and worsening MCID thresholds were applied to re-express effect sizes as probabilities of improvement, stability, and worsening. These probabilities were then expressed as absolute probability change, relative benefit, and odds updating, and their potential application to evidence interpretation and patient communication during the initial consultation was explored.

## Materials and methods

Study design

**Figure 1:**
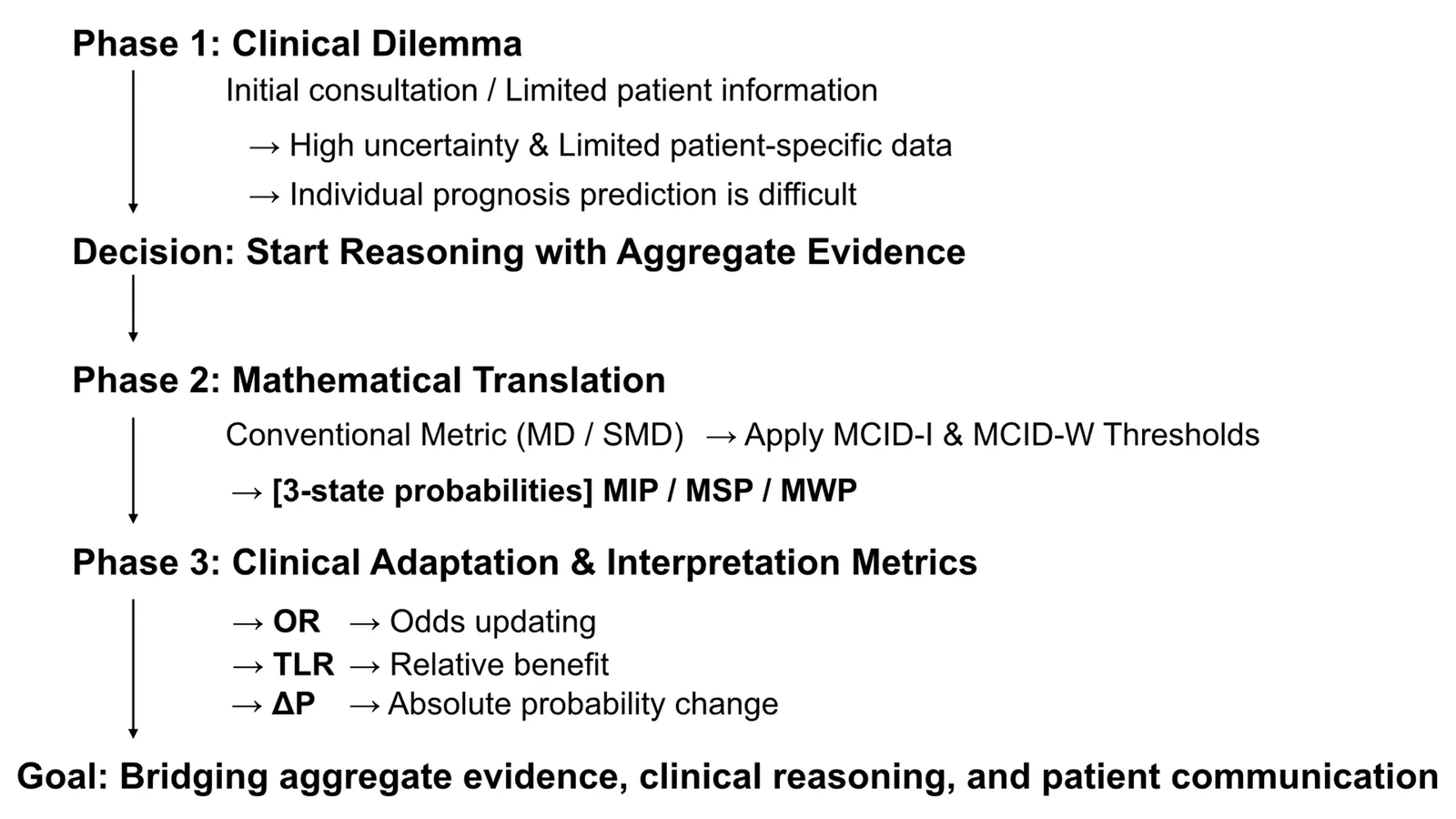
Conceptual framework for translating aggregate treatment effects into clinically interpretable probabilities. The overall aim is to bridge aggregate evidence and individual clinical interpretation while preserving the original effect-size information. This figure is intended to illustrate the conceptual workflow of the proposed framework rather than a direct clinical implementation. This image was created by the authors of this study. MD: mean difference; SMD: standardized mean difference; MCID: minimal clinically important difference; MCID-I: minimal clinically important difference-improvement; MCID-W: minimal clinically important difference-worsening; MIP: minimal clinically important difference-improvement probability; MSP: minimal clinically important difference-stability probability; MWP: minimal clinically important difference-worsening probability; TLR: therapeutic leverage ratio; ΔP: absolute probability difference

This exploratory study re-expressed effect sizes reported in randomized controlled trials and meta-analyses of acupuncture as clinically interpretable probabilities and examined their potential application to evidence interpretation and patient communication. The study consisted of three stages (Figure 1). First, effect sizes were re-expressed as probabilities of improvement, stability, and worsening using MCID thresholds. Second, these probabilities were interpreted through odds ratios (ORs) as a probability-updating process that may support clinicians’ interpretation of evidence. Third, the same probabilities were expressed as absolute probability differences (ΔP) and therapeutic leverage ratios (TLRs), representing absolute probability change and relative benefit, respectively, to explore their potential role in facilitating shared understanding and communication of treatment effects. These proposed roles were conceptually based on previous studies indicating that the presentation format of treatment effects influences understanding and decision-making, rather than on direct empirical evaluation within the present study [2,3]. Sensitivity analyses were also performed.

Data sources and effect sizes

We extracted mean differences (MDs) or standardized mean differences (SMDs) based on continuous outcomes, including pain, physical function, and exercise tolerance, from previously published randomized controlled trials and systematic reviews. Priority was given to studies that reported standard deviations (SDs), follow-up periods, comparator conditions, and MCID thresholds. No restrictions were placed on disease category, comparator type, acupuncture technique, stimulation method, or statistical significance of the reported results.

Studies were included when effect sizes, MCID thresholds, and SD values (or sufficient information to estimate or reconstruct SDs) could be obtained. Studies reporting only relative measures (e.g., RR or OR), studies with unclear outcome scales, conference abstracts, narrative reviews, protocols, and reports without sufficient information for probability conversion were excluded.

This study was not intended to be a systematic review or meta-analysis. Rather, it was conducted as an exploratory investigation to demonstrate potential applications of the present approach. Therefore, we did not apply Grading of Recommendations, Assessment, Development, and Evaluation (GRADE) assessments or preregistered review procedures. Instead, we included reports from which effect sizes and related parameters could be obtained. The purpose of this study was not to synthesize treatment effects but to examine the feasibility and interpretability of the proposed approach using published data.

Probability conversion

To prioritize simplicity and clinical interpretability, we used previously described methods for probability conversion [4,6]. The aim of this study was not individual prognosis prediction, but rather to enhance the clinical interpretability of population-level summary statistics and support evidence interpretation when patient-specific information is limited.

Effect sizes were analyzed under the assumption of a normal distribution. To maintain consistency with SMD-based representations, the mean of the control group was standardized to zero. The intervention-group mean was represented by the reported MD or SMD.

Next, MCID thresholds were defined for improvement (MCID-I) and worsening (MCID-W). Whenever available, anchor-based thresholds reported in previous studies were used [7-11]. When outcome measures differed across studies and a common threshold could not be defined, a distribution-based threshold (±0.5 SD) was used as a pragmatic reference value [6,12].

The probability of improvement was calculated as the area of the distribution above the MCID-I threshold. The probability of worsening was calculated as the area below the MCID-W threshold. The probability of stability was defined as the area between the improvement and worsening thresholds.

**Figure 2:**
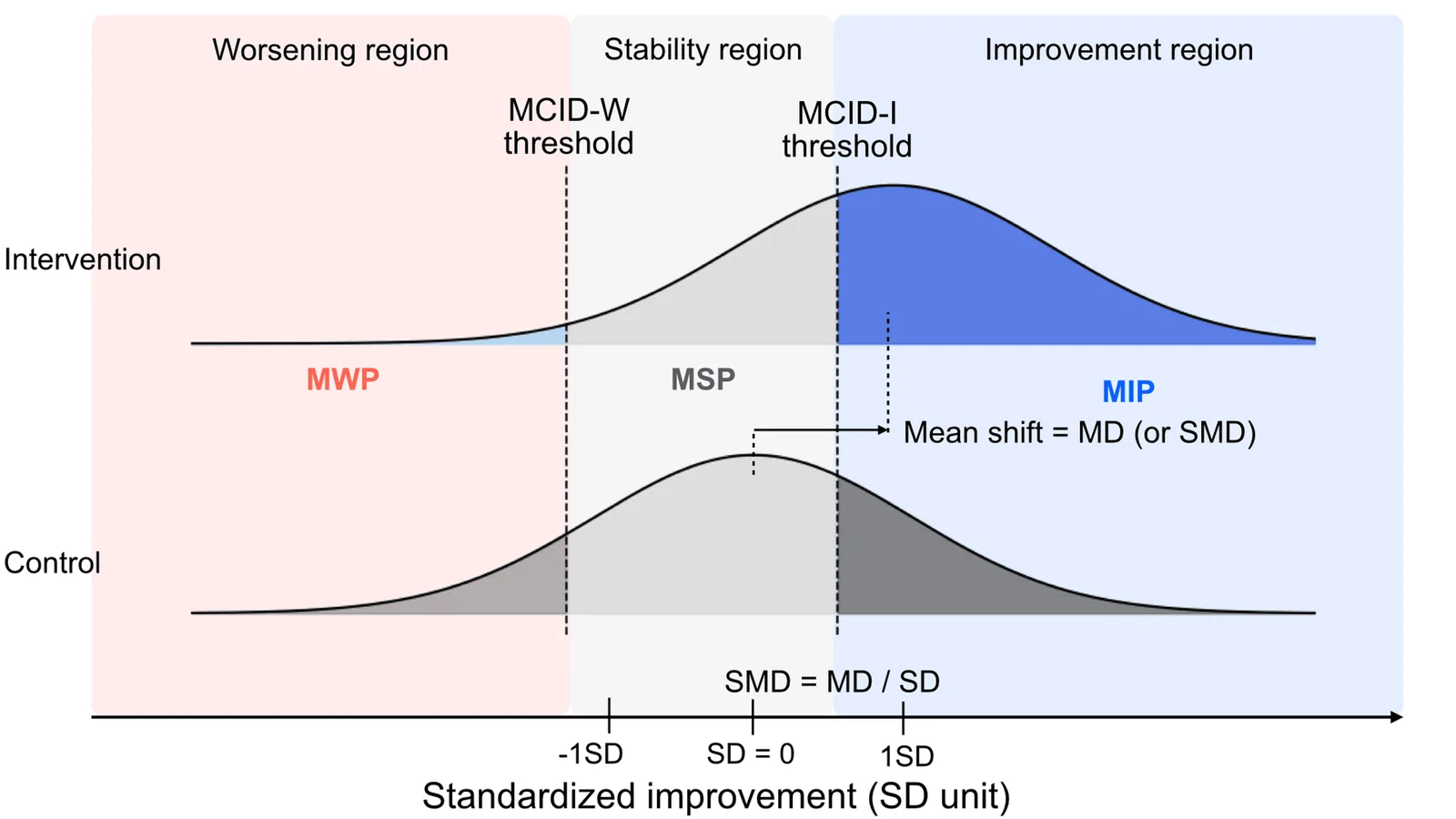
Re-expression of continuous treatment effects as probabilities of improvement, worsening, and stability outcomes. Continuous outcomes were assumed to follow normal distributions. Improvement and worsening thresholds were defined using MCID-I and MCID-W. The area beyond the improvement threshold represents the probability of clinically meaningful improvement, whereas the area below the worsening threshold represents the probability of clinically meaningful worsening. The area between the two thresholds represents the probability of stability. This framework illustrates how conventional effect measures (MD or SMD) can be translated into probabilities of improvement, worsening, and stability while preserving the underlying distribution. This image was created by the authors of this study from study data using Python and assembled using Keynote (Cupertino, CA: Apple Inc.). MCID: minimal clinically important difference; MCID-I: minimal clinically important difference-improvement; MCID-W: minimal clinically important difference-worsening; MD: mean difference; SMD: standardized mean difference; MIP: minimal clinically important difference-improvement probability; MWP: minimal clinically important difference-worsening probability; MSP: minimal clinically important difference-stability probability

For clarity, the probability of improvement based on MCID-I was termed MCID-improvement probability (MIP), the probability of worsening based on MCID-W was termed MCID-worsening probability (MWP), and the probability of stability was termed MCID-stability probability (MSP) (Figure 2). A detailed description of the computational procedures is provided in appendix 1.

Probability updating

Using previously described methods, odds and odds ratios (ORs) were calculated from the estimated probabilities. The probabilities derived for the control group were first converted into prior odds, which were then updated through ORs to obtain the corresponding post-test probabilities. In the present study, ORs were used as probability-updating measures that may support clinicians’ interpretation of evidence. This updating process was intended to represent how population-level baseline evidence may be adapted and interpreted during the initial consultation, where patient-specific information is often limited. Metrics based on improvement were denoted OR+; those based on worsening were denoted OR-; and those based on stability were denoted OR(S).

Communication metrics

The same probabilities were further expressed as TLRs and ΔP values. TLRs represented the relative benefit associated with treatment, whereas ΔP represented the absolute change in probability between intervention and control groups. In the present study, these measures were used as complementary formats for communicating treatment effects and facilitating shared understanding. Metrics based on improvement, worsening, and stability were denoted as TLR+, TLR-, and TLR(S), and ΔP+, ΔP-, and ΔP(S). Although TLR shares the same mathematical form as a risk ratio (RR), the term TLR was used to emphasize its interpretive role and to distinguish it from conventional RRs used for unavoidable events such as disease occurrence or mortality.

Sensitivity and statistical analysis

Because probability conversion depends on several assumptions, including distributional form, MCID thresholds, and effect size estimates, sensitivity analyses were conducted to examine the robustness of the approach. To quantitatively evaluate the relationships between the original effect sizes and the derived probability-based measures, Spearman’s rank correlation analyses were performed separately for the MD- and SMD-based examples. Correlations were examined for MIP, MWP, MSP, ΔP, and TLR. Odds ratios were not included because they additionally depend on baseline probabilities and therefore do not represent a direct transformation of the original effect sizes. Statistical analyses were performed using StatView version 5.0 (Cary, NC: SAS Institute Inc.). A two-sided p<0.05 was considered statistically significant.

The purpose of this study was not to test statistical hypotheses but to explore the feasibility and interpretability of re-expressing published effect sizes as clinically interpretable probabilities. Accordingly, the analyses were primarily descriptive and exploratory in nature.

## Results

Probabilistic re-expression of effect sizes

Previously reported MDs and SMDs were successfully re-expressed as probabilities of meaningful improvement (MIP), meaningful worsening (MWP), and meaningful stability (MSP) [13-25]. Compared with the control groups, the acupuncture groups showed higher MIP values, lower MWP values, and lower or comparable MSP values (Table 1). In several studies, MIP increased, and MWP decreased relative to the control group, whereas MSP remained largely unchanged. This pattern was observed in studies [14,16,17,22].

**Table 1:** Re-expression of reported effect sizes as probabilities of clinically meaningful improvement, worsening, and stability. ^a^The reported 95% confidence interval appeared inconsistent with the corresponding effect estimate and is presented as reported in the original publication [22]. ^b^MCID-I and MCID-W were derived from external reference values rather than COPD-specific MCIDs [19]. MD: mean difference; SMD: standardized mean difference; MCID: minimal clinically important difference; MCID-I: minimal clinically important difference-improvement; MCID-W: minimal clinically important difference-worsening; MIP: minimal clinically important difference-improvement probability; MWP: minimal clinically important difference-worsening probability; MSP: minimal clinically important difference-stability probability; TLR: therapeutic leverage ratio; COPD: chronic obstructive pulmonary disease; NSLBP: non-specific low back pain; NSMSK: non-specific musculoskeletal; OA: osteoarthritis; PD: Parkinson's disease; RA: rheumatoid arthritis; TTH: tension-type headache; 6MWT: 6-minute walk test; FMA: Fugl-Meyer Assessment; NRS: numerical rating scale; VAS: visual analog scale; WOMAC: Western Ontario and McMaster Universities Osteoarthritis Index

Studies	Condition	Effect size (MD/SMD: 95% CI)	MCID-I	MCID-W	SD	MIP: control (%)	MWP: control (%)	MSP: control (%)	MIP: acupuncture (%)	MWP: acupuncture (%)	MSP: acupuncture (%)
Mu et al. (2020) [13]	NSLBP (immediate)	MD=20.32 (16.14, 24.50)	VAS=15 mm [7]	VAS=-16.55 mm [8]	Pooled SD=20.51	23.2	21.0	55.8	60.2	3.6	36.2
Mu et al. (2020) [13]	NSLBP (short)	MD=10.11 (3.43, 16.80)	VAS=15 mm [7]	VAS=-16.55 mm [8]	Pooled SD=20.51	23.2	21.0	55.8	40.6	9.7	49.7
Manheimer et al. (2018) [14]	Hip OA	MD=2.16 (-7.91, 3.55)	WOMAC=1.85 points [9]	WOMAC=-1.4 points [9]	Pooled SD=16.2	45.5	46.6	7.9	50.8	41.3	7.9
Melchart et al. (2005) [15]	TTH	MD=1.6 (1.23, 10.11)	VAS=1.5 points [7]	VAS=-1.655 points [8]	Pooled SD=1.56	16.8	14.4	68.8	52.6	1.8	45.6
Ju et al. (2017) [16]	Neuropathic pain	MD=0.40 (-1.03, 1.83)	VAS=1.5 points [7]	VAS=-1.655 points [8]	Pooled SD=2.46	27.1	25.1	47.8	32.6	20.2	47.2
Manheimer et al. (2010) [17]	Peripheral joint OA	MD=0.92 (0.36, 1.48)	WOMAC=1.85 points [9]	WOMAC=-1.4 points [9]	Pooled SD=3.29	28.7	33.5	37.8	38.9	24.0	37.1
Casimiro et al. (2005) [18]	RA	MD=7.0 (-0.40, 14.40)	VAS=15 mm [7]	VAS=-16.55mm [8]	Pooled SD=13.88	14.0	11.7	74.4	28.2	4.5	67.3
Suzuki et al. (2012) [19]	COPD	MD=78.68 (54.16, 103.21)	6MWT=28 m [10]^b^	6MWT=-33 m [10]^b^	Pooled SD=49.30	28.5	25.2	46.3	84.8	1.2	14.0
Smith et al. (2020) [20]	Labor pain	MD=1.16 (0.81, 1.51)	VAS=1.5 points [7]	VAS=-1.655 points [8]	Pooled SD=1.15	9.6	7.5	82.9	38.4	0.7	61.9
Yang et al. (2016) [21]	Stroke	MD=6.16 (4.20, 8.11)	FMA=+5.25 points [11]	FMA=-5.25 points [11]	Pooled SD=9.0	28.0	28.0	44.0	54.0	10.2	35.8
Trinh et al. (2016) [22]	Neck disorders	SMD=0.23 (0.07, 0.20)^a^	0.5 SD [6,12]	-0.5 SD [6,12]	SD=1.0	30.9	30.9	38.2	39.4	23.3	37.3
Sun et al. (2023) [23]	Migraine	SMD=0.56 (0.38, 0.73)	0.5 SD [6,12]	-0.5 SD [6,12]	SD=1.0	30.9	30.9	38.2	52.4	14.5	33.1
Vickers et al. (2018) [24]	Chronic headache	SMD=0.56 (0.35, 0.76)	0.5 SD [6,12]	-0.5 SD [6,12]	SD=1.0	30.9	30.9	38.2	52.4	14.5	33.1
Vickers et al. (2018) [24]	NSMSK (back and neck)	SMD=0.50 (0.38, 0.63)	0.5 SD [6,12]	-0.5 SD [6,12]	SD=1.0	30.9	30.9	38.2	50.0	15.9	34.1
Vickers et al. (2018) [24]	OA	SMD=0.74 (0.46, 1.01)	0.5 SD [6,12]	-0.5 SD [6,12]	SD=1.0	30.9	30.9	38.2	59.5	10.8	29.7
Vickers et al. (2018) [24]	Specific shoulder pain	SMD=0.57 (0.44, 0.69)	0.5 SD [6,12]	-0.5 SD [6,12]	SD=1.0	30.9	30.9	38.2	52.8	14.2	33.0
Yu et al. (2025) [25]	PD	SMD=0.79 (0.49, 1.09)	0.5 SD [6,12]	-0.5 SD [6,12]	SD=1.0	30.9	30.9	38.2	61.4	9.9	28.7

Among the 10 conditions reported using MDs [13-21], effect sizes ranged from 0.40 in the study by Ju et al. to 78.68 in the study by Suzuki et al. [16,19]. However, when re-expressed as probabilities based on MCID thresholds, the conditions showing the highest and lowest MIP, MWP, and MSP values did not necessarily correspond to the ranking of the original effect sizes.

Among the seven conditions reported using SMDs [22-25], effect sizes ranged from 0.23 for neck disorders to 0.79 for Parkinson’s disease [22,25]. These conditions also corresponded to the minimum and maximum values of MIP, MWP, and MSP. However, higher MIP values were accompanied by lower MWP and MSP values, resulting in an inverse ranking across these probability measures.

Spearman’s rank correlation analyses were performed separately for the MD- and SMD-based examples. In the MD-based examples, the derived probability-based measures generally showed low-to-moderate correlations with the original effect sizes, and none reached statistical significance. The strongest correlations were observed for MIP (ρ=0.61, p=0.066), ΔP+ (ρ=0.59, p=0.078), and TLR- (ρ=0.56, p=0.090). In contrast, the SMD-based examples showed very strong monotonic correlations between the original effect sizes and the derived probability-based measures (ρ=0.96-1.00, all p≤0.018).

Probability updating and communication metrics

**Figure 3:**
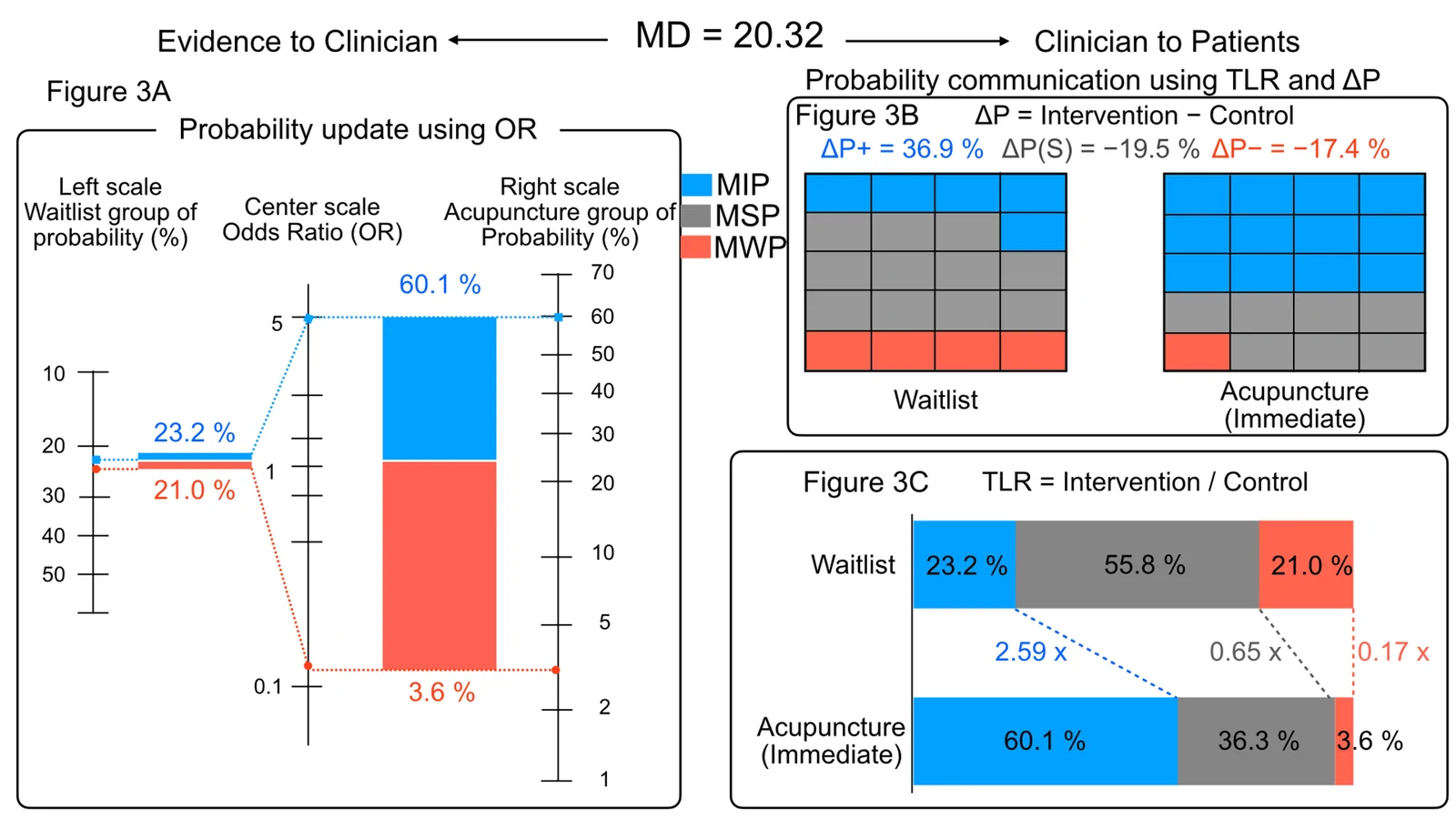
From probability estimates to evidence interpretation and communication. Probabilities derived from treatment effects can be interpreted from multiple perspectives. OR represents evidence interpretation through odds-based probability updating. TLR expresses the relative change in improvement probability between intervention and control groups, whereas ΔP expresses the absolute change in improvement probability. The example shown is based on immediate effects reported in non-specific low back pain and illustrates how the same probability information can be translated into different forms of evidence interpretation and communication [13]. This image was created by the authors of this study from study data using Python and assembled using Keynote (Cupertino, CA: Apple Inc.). MD: mean difference; MIP: minimal clinically important difference-improvement probability; MWP: minimal clinically important difference-worsening probability; MSP: minimal clinically important difference-stability probability; TLR: therapeutic leverage ratio; ΔP: absolute probability difference

Several characteristics were observed when probabilities were re-expressed using ORs, TLRs, and ΔP (Table 2). These metrics were derived from the same underlying probability data. For example, in the immediate-effect analysis of NSLBP reported by Mu et al., an increase in improvement probability from 23.2% to 60.2% corresponded to OR+=5.0 (Figure 3A), ΔP+=36.9% (Figure 3B), and TLR+=2.59 (Figure 3C) [13].

**Table 2:** Interpretation metrics derived from probability-based outcomes. ^a^The reported 95% confidence interval appeared inconsistent with the corresponding effect estimate; therefore, TLR 95% confidence intervals were not calculated [22]. ^b^TLR(s) confidence intervals were derived from the original effect-size confidence intervals and may not contain the point estimate because of the non-linear nature of stability probabilities [16]. TLR: therapeutic leverage ratio; ΔP: absolute probability difference

Studies	ΔP+ (%)	ΔP- (%)	ΔP(S) (%)	TLR+ (estimate 95% CI)	TLR- (estimate 95% CI)	TLR(S) (estimate 95% CI)	OR+	OR-	OR(S)
Mu et al. (2020) [13]	36.9	-17.4	-19.6	2.59 (2.25, 2.92)	0.17 (0.11, 0.26)	0.65 (0.52, 0.77)	5.0	0.14	0.45
Mu et al. 2020 [13]	17.3	-11.3	-6.0	1.75 (1.10, 2.85)	0.46 (0.28, 0.73)	0.89 (0.74, 0.98)	2.26	0.40	0.78
Manheimer et al. (2018) [14]	5.3	-5.3	0.0	1.12 (0.6, 1.19)	0.89 (0.82, 1.41)	1.00 (0.90, 1.0)	1.23	0.81	1.0
Melchart et al. (2005) [15]	35.7	-12.6	-23.2	3.13 (2.0, 4.80)	0.13 (0.11, 0.38)	0.66 (0.001, 0.78)	5.48	0.11	0.38
Ju et al. (2017) [16]	5.5	-4.9	-0.6	1.21 (0.6, 2.0)	0.81 (0.32, 1.60)	0.98 (0.95, 1.04)^b^	1.31	0.76	0.97
Manheimer et al. (2010) [17]	10.2	-9.5	-0.7	1.35 (1.13, 1.59)	0.72 (0.57, 0.88)	0.98 (0.94, 1.0)	1.58	0.63	0.97
Casimiro et al. (2005) [18]	14.2	-7.2	-7.1	2.01 (0.95, 3.45)	0.39 (0.11, 1.05)	0.91 (0.68, 1.0)	2.40	0.36	0.71
Suzuki et al. (2012) [19]	56.3	-24.0	-32.3	2.98 (2.46, 3.29)	0.05 (0.01, 0.15)	0.30 (0.11, 1.6)	14.10	0.03	0.19
Smith et al. (2020) [20]	28.8	-6.8	-22.0	3.96 (3.04, 4.94)	0.09 (0.05, 0.16)	0.74 (0.60, 0.85)	5.81	0.09	0.32
Yang et al. (2016) [21]	25.9	-17.8	-8.2	1.93 (1.63, 2.23)	0.36 (0.25, 0.51)	0.81 (0.70, 0.91)	3.0	0.29	0.72
Trinh et al. (2016) [22]	8.5	-7.6	-1.0	1.27^a^	0.76^a^	0.97^a^	1.46	0.68	0.95
Sun et al. (2023) [23]	21.5	-16.5	-5.1	1.70 (1.44, 2.07)	0.47 (0.33, 0.62)	0.87 (0.79, 0.94)	2.47	0.38	0.80
Vickers et al. (2018) [24]	21.5	-16.5	-5.1	1.70 (1.41, 2.12)	0.47 (0.31, 0.61)	0.87 (0.75, 0.94)	2.47	0.38	0.80
Vickers et al. (2018) [24]	19.1	-15.0	-4.1	1.62 (1.47, 1.79)	0.52 (0.42, 0.61)	0.89 (0.84, 0.94)	2.23	0.43	0.83
Vickers et al. (2018) [24]	28.6	-20.1	-8.5	1.93 (1.57, 2.25)	0.35 (0.21, 0.55)	0.78 (0.63, 0.91)	3.28	0.27	0.68
Vickers et al. (2018) [24]	21.9	-16.7	-5.2	1.71 (1.54, 1.86)	0.45 (0.38, 0.56)	0.87 (0.81, 0.92)	2.49	0.35	0.81
Yu et al. (2025) [25]	30.5	-21.0	-9.5	1.99 (1.66, 2.32)	0.32 (0.18, 0.48)	0.75 (0.53, 0.91)	3.56	0.25	0.65

Across all conditions, OR+ values were consistently larger than the corresponding TLR+ values (Table 3). The OR+/TLR+ ratio ranged from 1.08 in neuropathic pain to 4.73 in chronic obstructive pulmonary disease (COPD) [16,19]. ΔP+ values ranged from 5.5% to 56.3%, whereas TLR+ values ranged from 1.12 to 3.96.

**Table 3:** Relationship between ΔP+, TLR+, and OR+ across included conditions. TLR: therapeutic leverage ratio; ΔP: absolute probability difference; COPD: chronic obstructive pulmonary disease; NSLBP: non-specific low back pain; NSMSK: non-specific musculoskeletal; OA: osteoarthritis; PD: Parkinson's disease; RA: rheumatoid arthritis; TTH: tension-type headache

Condition	ΔP+ (%)	TLR+	OR+	OR+/TLR+
Neuropathic pain [16]	5.5	1.21	1.31	1.08
Hip OA [14]	5.3	1.12	1.23	1.10
Neck disorders [22]	8.5	1.27	1.46	1.15
Peripheral OA [17]	10.2	1.35	1.58	1.17
RA [18]	14.2	2.01	2.40	1.19
NSLBP (short) [13]	17.3	1.75	2.26	1.29
NSMSK (back and neck) [24]	19.1	1.62	2.23	1.38
Migraine [23]	21.5	1.70	2.46	1.45
Chronic headache [24]	21.5	1.70	2.46	1.45
Specific shoulder pain [24]	21.9	1.71	2.49	1.46
PD [25]	30.5	1.99	3.56	1.46
Labor pain [20]	28.8	3.96	5.81	1.47
Stroke [21]	25.9	1.93	3.00	1.55
OA [24]	28.6	1.93	3.28	1.70
TTH [15]	35.7	3.13	5.48	1.75
NSLBP (immediate) [13]	36.9	2.59	5.0	1.93
COPD [19]	56.3	2.98	14.10	4.73

The relationship between ΔP and TLR varied across conditions. For example, neuropathic pain and COPD showed approximately a tenfold difference in ΔP+ (5.5% vs. 56.3%), whereas the corresponding TLR+ values differed by less than threefold (1.21 vs. 2.98) [16,19]. In contrast, Parkinson’s disease and labor pain showed similar ΔP+ values (30.5% vs. 28.8%) but different TLR+ values (1.99 vs. 3.96) [20,25].

In the three studies that did not report statistically significant effects (hip osteoarthritis, neuropathic pain, and rheumatoid arthritis), the estimated 95% confidence intervals of TLR crossed 1 [14,16,18]. In addition, one study reported a TLR(S) point estimate that was not contained within its estimated 95% confidence interval [16].

Sensitivity analysis

Sensitivity analyses were conducted by varying MCID thresholds, effect size estimates, baseline probabilities, and distributional assumptions (appendix 2). When MCID thresholds became more stringent, MIP decreased, whereas TLR+ and OR+ increased. Correspondingly, MWP decreased, while MSP increased. Increasing effect sizes resulted in higher MIP values and lower MWP and MSP values, with TLR and OR generally changing in the same direction as underlying probabilities.

Updated probabilities also varied according to baseline probability despite identical OR values. For example, under an SMD of 0.56 and an OR+ of 2.47, the probability of improvement increased from 20.0% to 38.1% when the baseline probability was 20%, whereas it increased from 40.0% to 62.2% when the baseline probability was 40%.

Alternative distributional assumptions influenced the estimated probabilities. MIP was more sensitive to changes in distribution skewness, whereas MWP was more affected by changes in distribution width. MSP varied under both conditions. Despite these changes in absolute probabilities and interpretation metrics, the overall three-category structure of improvement, worsening, and stability was preserved across all scenarios.

## Discussion

Overall interpretation

The present study was not intended to propose a new statistical measure or prediction model. Rather, its distinctive feature lies in integrating existing concepts and methods into a framework for clinical interpretation. MDs, SMDs, MCIDs, probabilities, ORs, TLRs, and ΔP are all established concepts. Although each computational component has been reported previously, we were unable to find reports that describe this integrated calculation process as a unified framework for translating effect sizes into clinically interpretable probabilities. These concepts are often used independently and are rarely considered as part of a continuous process linking average treatment effects to patient-level interpretation.

An additional feature of the present approach is that three outcome states, improvement, worsening, and stability, can be derived simultaneously from a single effect size. Furthermore, the same probability estimates can be expressed using different interpretation metrics according to purpose, including ORs, TLRs, and ΔP. To our knowledge, few reports have examined the probabilities of improvement, worsening, and stability together with multiple interpretation metrics within a unified framework. By integrating these existing concepts without introducing new statistical measures, we explored an approach that may help bridge the gap between average treatment effects and individual patients.

Additional Spearman’s rank correlation analyses demonstrated generally low-to-moderate correlations between the original effect sizes and the derived probability-based measures in the MD-based examples, whereas very strong monotonic correlations were observed in the SMD-based examples. The lower correlations in the MD-based examples likely reflect variation in study-specific standard deviations, whereas the standardized variance inherent to SMD-based analyses preserved the rank order of the derived probability-based measures. These findings suggest that probability-based measures derived from MDs are influenced not only by the magnitude of the original effect size but also by study-specific variability, which may partly explain why metrics such as TLR provide complementary information regarding the relative position of treatment effects.

From distributions to probabilities

Converting effect sizes into probabilities using MCID thresholds may facilitate clinical interpretation of treatment effects [1,4,5]. Rather than expressing treatment effects solely as mean differences or standardized mean differences, this approach allows outcomes to be interpreted as probabilities. Such probabilities may help clinicians interpret population-level evidence and communicate expected treatment outcomes to patients when patient-specific information is limited.

An important feature of this framework is that it allows improvement, worsening, and stability to be evaluated simultaneously from the same effect size. Treatment effects are often discussed primarily in terms of improvement. However, considering worsening and stability alongside improvement may provide additional information relevant to clinical interpretation, including the balance between potential benefits and risks. Although MSP is mathematically derived from the same probability distribution as MIP and MWP, reporting all three outcome states provides a more comprehensive description of treatment effects than improvement alone. In the present study, some conditions showed little change in MSP despite increases in MIP and decreases in MWP. These findings suggest that considering improvement, worsening, and stability together may provide complementary information for interpreting treatment effects, although the clinical significance of these patterns requires further investigation.

Improvement and worsening thresholds were defined using anchor-based MCID thresholds whenever available. When outcome measures differed across studies and a common threshold could not be established, a distribution-based MCID of 0.5 SD was used. Anchor-based thresholds were prioritized whenever possible.

The use of a fixed 0.5 SD threshold or a population-average effect size as a direct predictor for individual patients has been criticized [26]. However, when patient-specific information is limited, clinicians may lack sufficient information on individual prognostic factors. Under such circumstances, the 0.5 SD benchmark proposed by Norman et al., together with average effect sizes derived from meta-analyses, may serve as practical starting points for interpreting treatment effects [12]. The key issue is not whether anchor-based or distribution-based MCIDs should be used universally, but how each approach can be applied according to the available information and the purpose of interpretation.

From probabilities to interpretation metrics

In the present study, three complementary metrics, OR, TLR, and ΔP, were derived from the same underlying probability estimates. As illustrated in Figure 3, these measures may function as separate but complementary tools within the pathway from evidence interpretation to communication. Although this hypothesis was not empirically evaluated, clinicians and patients may benefit from different forms of treatment-effect expression. For this reason, multiple interpretation metrics were presented together.

OR functions as a probability-updating measure in a manner analogous to likelihood ratios [27]. By treating the probability observed in the control group as a prior probability, OR can be used to express how the probability of improvement or worsening changes following acupuncture. This probability-updating process also conceptually resembles Bayesian reasoning, in which prior probabilities are updated by new evidence to estimate posterior probabilities. Although the present framework does not implement a Bayesian statistical model, this similarity may provide a useful conceptual bridge between evidence synthesis and probabilistic clinical reasoning. Although it is not intended as a formal individual prognostic model, it may support clinicians’ interpretation of evidence during the initial consultation, when patient-specific information is often limited. At the same time, OR has an important limitation as follows: it is often difficult to interpret intuitively [28-30]. In particular, when event rates are high, ORs are known to exaggerate the apparent magnitude of relative effects. Consistent with this observation, ORs and TLRs derived from the same probabilities produced different numerical values in the present study.

For this reason, alternative expressions may be useful when communicating treatment effects to patients. Relative measures, such as TLR, which indicate how many times a probability changes, and absolute measures, such as ΔP, which indicate how much a probability increases or decreases, may be easier to communicate and understand. The present findings showed that similar ΔP values could be associated with different TLR values, and vice versa. This suggests that relative and absolute probability measures may capture different aspects of treatment-effect interpretation. TLR and ΔP may therefore provide complementary perspectives on the same probability shift and may help facilitate shared understanding between clinicians and patients.

Taken together, OR may be particularly useful for evidence interpretation by clinicians, whereas TLR and ΔP may provide complementary formats for communicating treatment effects and facilitating shared understanding with patients. Although the present study did not evaluate users’ understanding of ORs, TLRs, or ΔP, previous studies have shown that probability-based presentations can improve the understanding of treatment effects [2,3]. Future studies should therefore evaluate how these interpretation metrics are perceived by both clinicians and patients. In actual clinical practice, clinicians first obtain patient-specific information through history taking and clinical examination before applying evidence to individual patients. Accordingly, the present framework is intended to complement, rather than replace, this clinical reasoning process.

Limitations

This study has several limitations. First, the probability estimates were derived from summary statistics and therefore depended on a series of assumptions, including the use of normal distributions, available SD values, and selected MCID thresholds. These assumptions may not fully reflect the distributions and clinical characteristics observed in real-world patient populations. This issue may be particularly relevant in studies such as the COPD study, where measurement ranges and SDs were relatively large and could influence probability and odds estimates [19]. The results should therefore be interpreted in light of the characteristics of the outcome measures. However, the purpose of the present study was not to provide precise individual predictions but to re-express population-level evidence into a clinically interpretable form. From this perspective, these assumptions may serve as practical starting points for evidence interpretation when patient-specific information is limited.

Second, future work should explore alternative probability models, including truncated normal distributions and other approaches for bounded outcome scales. Third, this was an exploratory study and was not conducted as a systematic review. The aim was not to synthesize intervention effects but to examine the feasibility and interpretability of the proposed approach using published data.

Fourth, the probabilities presented in this study were derived from population-level summary statistics and are not intended to provide accurate predictions for individual patients. In addition, for studies in which SDs were reconstructed from published summary data, the reconstructed SDs may introduce additional uncertainty into the derived probability estimates. Future studies should examine baseline risk adjustment, comparisons with individual patient data, and the usefulness of ORs, TLRs, and ΔP for evidence interpretation and patient communication. Finally, the present approach was explored using acupuncture research. Further studies are needed to determine whether similar interpretations can be applied to other therapeutic interventions.

## Conclusions

This study re-expressed continuous outcomes (MDs and SMDs) as probabilities of improvement, worsening, and stability and explored their clinical interpretability. Previously reported effect sizes could be translated into probability-based outcomes and further interpreted using ORs, TLRs, and ΔP. Probabilistic re-expression may provide a more intuitive way to understand population-level evidence and help address the interpretive gap between average treatment effects and individual patients.

Although this approach is not intended for individual prediction, it provides an exploratory framework for interpreting published effect sizes as clinically meaningful probabilities. Its potential role in evidence interpretation, communication, and shared understanding should be evaluated in future studies using individual patient data and across different interventions, outcome measures, and clinical settings.

## Appendices

Appendix 1: computational procedures

The procedure for converting effect sizes into probabilities, and subsequently into ORs, TLRs, and ΔP, is illustrated using the immediate-effect study by Mu et al. [13]. The reported effect size was MD=20.32 with SD=20.51. The outcome measure was a 0-100 mm visual analog scale (VAS), and the MCID thresholds were 15.0 mm for improvement (MCID-I) and -16.55 mm for worsening (MCID-W). Probability conversion was based on a normal distribution. The control-group mean was fixed at zero, and the intervention-group mean was represented by the reported effect size. Z scores were calculated using the following formula.

$Z = \frac{\mathrm{threshold} - \mathrm{mean}}{\mathrm{SD}}$

Probabilities were then obtained from the cumulative distribution function of the standard normal distribution. Improvement probability (MIP) was calculated as 1 - Φ(Z_improvement_), worsening probability (MWP) as Φ(Z_worsening_), and stability probability (MSP) as 1 - MIP - MWP. Using the data reported by Mu et al., MIP increased from 23.2% in the control group to 60.2% in the intervention group. MWP decreased from 21.0% to 3.6%, whereas MSP decreased from 55.8% to 36.2% [13]. These probabilities were subsequently converted into ORs, TLRs, and ΔP. Odds ratios were calculated using standard odds conversion formulas. TLR was calculated as the ratio of intervention-group probability to control-group probability, and ΔP was calculated as the absolute difference between intervention-group and control-group probabilities. These probabilities yielded OR+=5.01, OR-=0.14, and OR(S)=0.45; TLR+=2.59, TLR-=0.17, and TLR(S)=0.65; and ΔP+=+36.9%, ΔP-=-17.4%, and ΔP(S)=-19.6%.

When SD values were unavailable, pooled SD values were reconstructed from published summary statistics. For studies reporting standardized mean differences, pooled SD was estimated as SD=MD/SMD. The present approach assumes an unbounded normal distribution. Future studies may explore alternative probability models, including truncated normal distributions and other methods that explicitly account for bounded outcome scales.

Excel Formulas

Improvement probability (MIP) = 1-NORM.S.DIST(Z_improvement,TRUE), Worsening probability (MWP) = NORM.S.DIST(Z_worsening,TRUE), and Stability probability (MSP) = 1-MIP-MWP

Appendix 2: sensitivity analyses

Sensitivity analyses were conducted to examine the influence of key assumptions on the probability-based framework. These analyses included variations in MCID thresholds, effect sizes, baseline probabilities, and distributional assumptions (distribution skewness and distribution width). The corresponding numerical results are presented in Tables 4-7.

**Table 4:** Sensitivity analyses using anchor-based and distribution-based MCID thresholds. Table A-1: low=MCID-I: 1.00 points and MCID-W: -1.30 points, base=MCID-I: 1.50 points and MCID-W: -1.655 points, high=MCID-I: 2.00 points and MCID-W: -2.015 points. Table A-2: low=MCID-I: 21.7 m and MCID-W: -24.0 m, base=MCID-I: 28.0 m and MCID-W: -33.0 m, high=MCID-I: 37.0 m and MCID-W: -45.0 m. Table B: low=±0.3SD, base=±0.5SD, high=±0.7SD. MCID-I: minimal clinically important difference-improvement; MCID-W: minimal clinically important difference-worsening; MIP: minimal clinically important difference-improvement probability; MSP: minimal clinically important difference-stability probability; MWP: minimal clinically important difference-worsening probability; TLR: therapeutic leverage ratio; ΔP: absolute probability difference; COPD: chronic obstructive pulmonary disease

Table A. Anchor-based MCID thresholds												
Scenario	MIP (control/acupuncture) %	MWP (control/acupuncture) %	MSP (control/acupuncture) %	ΔP+ (%)	ΔP- (%)	ΔP(S) (%)	TLR+	TLR-	TLR(S)	OR+	OR-	OR(S)
Table A-1: labor pain [20]												
Low threshold	19.2/55.5	12.9/1.6	67.6/42.8	36.3	-11.3	-24.8	2.89	0.12	0.63	5.21	0.11	0.36
Base threshold	9.6/38.4	7.5/0.7	82.9/60.9	28.8	-6.8	-22.0	4.0	0.09	0.73	5.81	0.09	0.32
High threshold	4.2/23.4	4.1/0.3	91.8/76.3	19.2	-3.8	-15.5	5.60	0.07	0.83	7.0	0.07	0.29
Table A-2: COPD [19]												
Low threshold	33.0/87.6	31.3/1.9	35.7/10.5	54.6	-29.4	-25.19	2.65	0.06	0.29	14.34	0.043	0.21
Base threshold	28.5/84.8	25.2/1.2	46.3/14.0	56.3	-24.0	-32.3	2.97	0.047	0.30	13.99	0.035	0.19
High threshold	22.7/80.1	18.1/0.6	59.2/19.3	57.4	-17.5	-39.9	3.53	0.03	0.33	13.71	0.027	0.17
Table B. Distribution-based MCID thresholds												
MCID threshold	MIP (control/acupuncture) %	MWP (control/acupuncture) %	MSP (control/acupuncture) %	ΔP+ (%)	ΔP- (%)	ΔP(S) (%)	TLR+	TLR-	TLR(S)	OR+	OR-	OR(S)
Table B-1: chronic headache [24]												
±0.3SD	38.2/60.3	38.2/19.5	23.6/20.3	22.0	-18.7	-3.3	1.58	0.51	0.86	2.45	0.39	0.82
±0.5SD	30.9/52.4	30.9/14.5	38.3/33.2	21.5	-16.4	-5.1	1.70	0.47	0.87	2.47	0.38	0.80
±0.7SD	24.2/44.4	24.2/10.4	51.6/45.2	20.2	-13.8	-6.4	1.84	0.43	0.88	2.51	0.36	0.77
Table B-2: neck disorders [22]												
±0.3SD	38.2/47.2	38.2/29.8	23.6/23.0	9.0	-8.4	-0.6	1.24	0.78	0.97	1.45	0.69	0.97
±0.5SD	30.9/39.4	30.9/23.3	38.2/37.3	8.5	-7.6	-0.9	1.28	0.75	0.98	1.45	0.68	0.96
±0.7SD	24.2/31.9	24.2/17.6	51.6/50.5	7.7	-6.6	-1.1	1.32	0.73	0.98	1.47	0.67	0.96
Table B-3: osteoarthritis [25]												
±0.3SD	38.2/67.0	38.2/14.9	23.6/18.1	28.8	-23.3	-5.5	1.75	0.39	0.77	3.28	0.28	0.72
±0.5SD	30.9/59.5	30.9/10.8	38.2/29.7	28.6	-20.1	-8.5	1.93	0.35	0.78	3.29	0.27	0.68
±0.7SD	24.2/51.6	24.2/7.5	51.6/40.9	27.4	-16.7	-10.7	2.13	0.31	0.79	3.34	0.25	0.65

**Table 5:** Sensitivity analyses across effect-size magnitudes. MIP: minimal clinically important difference-improvement probability; MSP: minimal clinically important difference-stability probability; MWP: minimal clinically important difference-worsening probability; TLR: therapeutic leverage ratio; ΔP: absolute probability difference; COPD: chronic obstructive pulmonary disease

Scenario	Effect size	MIP (control/acupuncture) %	MWP (control/acupuncture) %	MSP (control/acupuncture) %	ΔP+ (%)	ΔP- (%)	ΔP(S) (%)	TLR+	TLR-	TLR(S)	OR+	OR-	OR(S)
Table A. Distribution-based interpretation ranges: chronic headache [24]													
Lower effect	0.35	30.9/44.0	30.9/19.8	38.3/36.2	13.2	-11.1	-2.1	1.43	0.64	0.95	1.76	0.55	0.91
Point estimate	0.56	30.9/52.4	30.9/14.5	38.3/33.2	21.5	-16.4	-5.1	1.70	0.47	0.87	2.47	0.38	0.80
Upper effect	0.76	30.9/60.3	30.9/10.4	38.3/29.4	29.4	-20.5	-8.9	1.95	0.34	0.77	3.40	0.26	0.67
Table B. Anchor-based interpretation ranges: COPD [19]													
Lower effect	54.16	28.5/70.2	25.2/3.9	46.3/25.9	41.7	-21.3	-20.4	2.46	0.15	0.56	5.91	0.12	0.41
Point estimate	78.68	28.5/84.8	25.2/1.2	46.3/14.0	56.3	-24.0	-32.3	2.97	0.047	0.30	13.99	0.035	0.19
Upper effect	103.21	28.5/93.6	25.2/0.3	46.3/6.1	65.1	-24.9	-40.2	3.28	0.012	0.13	36.70	0.009	0.076

**Table 6:** Sensitivity analysis across baseline probabilities. Assumptions: OR+=2.47; OR-=0.38

Baseline scenario	Prior probability	Prior odds	Posterior odds	Updated probability
Low baseline improvement	20.0%	0.250	0.617	38.1%
Average baseline improvement	30.9%	0.446	1.100	52.4%
High baseline improvement	40.0%	0.667	1.644	62.2%
Low baseline worsening	20.0%	0.250	0.095	8.6%
Average baseline worsening	30.9%	0.446	0.169	14.5%
High baseline worsening	40.0%	0.667	0.253	20.2%

**Table 7:** Sensitivity analysis under alternative distributional assumptions. Skewed distributions are schematic examples standardized to the same mean shift and SD scale. These scenarios illustrate how distributional assumptions can alter probability-based interpretation even when the mean effect remains unchanged. MIP: minimal clinically important difference-improvement probability; MSP: minimal clinically important difference-stability probability; MWP: minimal clinically important difference-worsening probability

Distribution scenario	MIP (control) %	MIP (acupuncture) %	MWP (control) %	MWP (acupuncture) %	MSP (control) %	MSP (acupuncture) %
Normal	30.9	52.4	30.9	14.5	38.2	33.1
Narrow distribution	23.8	53.4	23.8	6.5	52.4	40.1
Wide distribution	35.0	51.8	35.0	20.7	30.0	27.5
Right-skewed	27.5	46.2	35.5	12.6	37.0	41.2
Left-skewed	35.5	58.3	27.5	14.8	37.0	26.9
